# Sodium Benzoate as an Emerging but Problematic Allergen: Retrospective Analysis of Patch Test Results in 3198 Cases Underlines the Need for an Improved Test Preparation, as Even Dubious Reactions May Be Clinically Relevant

**DOI:** 10.1111/cod.14803

**Published:** 2025-05-07

**Authors:** Nicholas J. Lawrance, Catherine R. Holden, David J. Gawkrodger

**Affiliations:** ^1^ Department of Dermatology Royal Hallamshire Hospital Sheffield UK; ^2^ Department of Dermatology University of Sheffield Sheffield UK

**Keywords:** allergic contact dermatitis, allergic reaction, clinical relevance, irritant reaction, preservative, sodium benzoate

## Abstract

**Background:**

Sodium benzoate is increasingly used as a preservative in personal care products and in foods. Recently published experience of patch testing sodium benzoate at 5% in pet shows it to be an allergen that may be assuming increasing clinical relevance.

**Objectives:**

We set out to define the prevalence of clinically relevant reactions to sodium benzoate, to delineate problems with the interpretation of patch test readings to this allergen and to identify whether its inclusion in various series offers additional diagnostic benefit beyond those offered by commonly tested allergens, for example benzoic acid and *Myroxylon pereirae* , with which it may show some cross reactivity.

**Methods:**

From 2008 to 2023, 3198 patients were patch tested to 5% sodium benzoate in pet, which has been included in our bakery/cheilitis, fragrance and facial series. Types of reaction, clinical relevance and cross‐reactivities were noted.

**Results:**

Of 3198 subjects tested to 5% sodium benzoate in pet, 57 (1.8%) had an allergic positive reaction (+/++), 53 (1.6%) had a doubtful reaction (?+) and 133 (4.1%) had an irritant reaction (IR) – meaning that ?+ or irritant reactions were more than three times more common than allergic responses. Clinical relevance was identified in 67% (38 of 57) of + or ++ reactors to sodium benzoate, and in 36% (19 of 53) of those with doubtful (?+) reactions.

Co‐reactivity to benzoic acid was seen in 15.4% of cases + or ++ to sodium benzoate.

The positivity ratio (the proportion of + reactions compared to ++ and +++) was 96.5% and the reaction index (number of allergic positives compared to ?+ or IR) was −0.53. Both indices indicate a problematic allergen.

**Conclusions:**

This series reaffirms that sodium benzoate is an important allergen which should be included in bakery/cheilitis, fragrance and facial series in addition to benzoic acid and *Myroxylon pereirae*, with which it occasionally cross‐reacts. However, it is tricky to test and its reactions are difficult to interpret—underlining the need to refine the preparation used for patch testing.

## Graphical abstract

Sodium benzoate is emerging as a preservative found most frequently in personal care products. It was positive in around 1.8% of a large series and should be included in the cheilitis/bakery, facial and fragrance patch test allergen series. As 5% sodium benzoate in pet it tends to cause false positive irritant reactions and true allergic responses are often weak, at + or at most ++ (Figure). Two‐thirds of allergic positives though are relevant clinically, and a third of ?+ reactions.

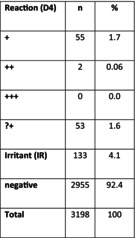

## 1 Introduction

**FIGURE 1:**
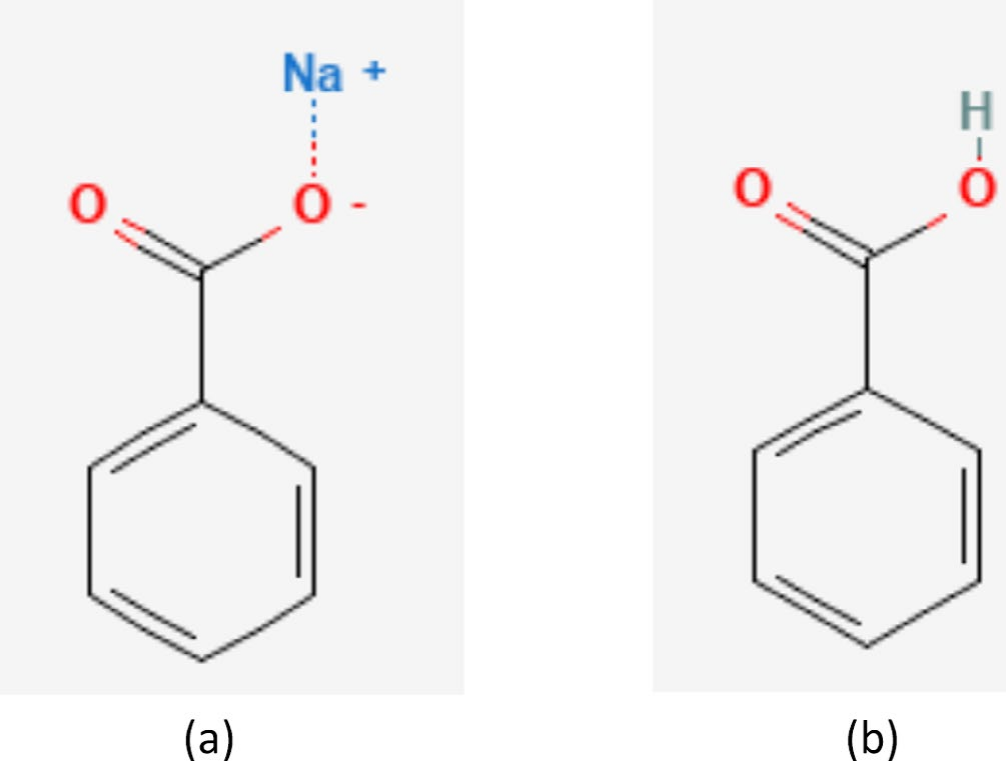
(a) Sodium benzoate, (b) benzoic acid.

Sodium benzoate (CAS Number: 532‐32‐1; EC Number: 208‐534‐8) is a naturally occurring preservative employed in the manufacturing and processing of formulations such as personal care products and foods [1, 2]. Its use has increased because of contact allergic problems and legislative restrictions associated with other preservatives, notably methylchloroisothiazolin‐one and methylisothiazolin‐one [3]. Sodium benzoate is a salt of benzoic acid and forms this when dissolved in water (Figure 1) [4, 5]. Benzoic acid is an oxidation metabolite of benzyl alcohol and a minor constituent of *Myroxylon pereirae* . Extensive experience has shown sodium benzoate as a food additive has low toxicity at permitted levels [2]. At high doses it may have potential for treating psychiatric conditions [6]. Sodium benzoate is rapidly absorbed through the skin, particularly when the barrier is damaged [7]. Previous studies have identified the irritant potential of sodium benzoate on patch testing but have not looked in detail at the relevance of reactions nor aspects of cross reactivity [8].

European Union legislation permits the use of sodium benzoate as a preservative at up to 0.5% (5000 ppm) for leave‐on products, at 2.5% (25 000 ppm) in rinse‐off preparations and 1.7% (17 000 ppm) in oral formulations [9]. The British Society for Cutaneous Allergy recently recommended the addition of 5% sodium benzoate in pet to its facial series, finding an allergic positive rate of 1.5%—their minimum for inclusion, following most international practice, being 0.5% to 1.0% (this publication did not mention irritant or dubious reaction rates) [10, 11].

In this report, we aim to review our experience with patch testing to sodium benzoate 5% in pet regarding types of reaction, problems in interpreting clinical relevance, its cross‐reactivities, and its suitability for inclusion in allergen patch test series.

## 2 Materials and Methods

### 2.1 Subjects

Between 2008 and 2023, 3198 adult and paediatric patients were patch tested to 5% sodium benzoate in pet, as a constituent of the bakery and cheilitis (2008–23), fragrance (2012–18) and facial (2015–18, 2021–23) series—applied when clinically indicated—at our tertiary contact dermatitis clinic. Concomitant allergic positive reactions to 5% benzoic acid in pet, 25% *Myroxylon pereirae* in pet and 10% benzyl alcohol in sof were noted. The ‘male, occupational, atopic, hand, leg, facial, age over 40 (MOAHLFA) index was recorded.

### 2.2 Patch Testing

Patch testing was performed in line with the guidelines of the European Society of Contact Dermatitis [11]. Materials (supplied by Chemotechnique MB Diagnostics AB, Vellinge, Sweden) were applied using Finn Chambers on Scanpor on the upper back (and in a small number of cases on the anterior thighs). Patches were removed on day 2 and read, with an additional reading on day 4 (some readings were at days 3 and 5).

### 2.3 Data Collection and Analysis

Statistical analysis was conducted using JASP (University of Amsterdam, the Netherlands). The reaction index—the proportion of allergic compared to irritant (IR) or doubtful (?+) reactions—and the positivity ratio—the proportion of + compared to ++ or +++ reactions—were calculated, to help identify if the reactions observed on patch testing with sodium benzoate were ‘problematic’. The range of the reaction index is from 1, if all reactions are +, ++ or +++ with no irritant or doubtful reactions, to −1 where reactions are only IR or ?+. Potential problems with the reading or interpretation of test results can be highlighted by a reactive index of less than zero, and by a positivity ratio of greater than 80% [8, 12].

## 3 Results

### 3.1 Baseline Characteristics

Just over 60% of allergic positive or doubtful positive reactors were female, less than 20% were deemed occupational, over three‐quarters were atopic, the majority of allergic positive individuals were over 40 years of age, and the commonest body site affected was the face—in just under a half (Table 1).

**TABLE 1:** Patch testing data for sodium benzoate 5% in pet, including MOAHLFA index, allergic positive reactions (+ or ++) and doubtful reactions (?+) but not irritant (IR) as at the day 4 reading.

Characteristics	Number (percent)		
	All patients	+ or ++	?+
Male	813/3101 (26.0%)	21/57 (36.8%)	20/51 (39.2%)
Occupational	365/2661 (13.7%)	9/57 (15.8%)	9/46 (19.6%)
Atopic	2192/3164 (69.3%)	44/57 (77.2%)	43/51 (84.3%)
Hand	705/3196 (22.1%)	14/57 (24.6%)	15/53 (28.3%)
Leg	66/3196 (2.1%)	1/57 (1.8%)	0/53 (0%)
Face	1493/3196 (46.7%)	26/57 (45.6%)	25/53 (47.2%)
Age over 40	2061/3198 (64.4%)	33/57 (57.9%)	30/53 (56.6%)

### 3.2 Contact Allergic Reactions to Sodium Benzoate

No +++ reactions were seen—most allergic reactions (55%–1.7%) were + with only two (0.06%) being of a ++ nature (Table 2). Doubtful (?+) reactions were apparent in 53 subjects (1.6%) with 133 (4.1%) showing irritant responses. The positivity rates differed between the various series tested, reflecting differences in indications for testing—the frequency of allergic + or ++ responses being as follows: bakery/cheilitis 1.5% (of 477 tested), facial 1.7% (1989 tested) and fragrance 2.1% (732 tested). The positivity ratio of 96.5% and the reactivity index of −0.53 indicate 5% sodium benzoate in pet to be a ‘problematic’ patch test preparation.

**TABLE 2:** Types of allergic, doubtful and irritant reactions as read on day 4, on patch testing with 5% sodium benzoate in pet.

Reaction (D4)	*n*	%
+	55	1.7
++	2	0.06
+++	0	0.0
?+	53	1.6
Irritant (IR)	133	4.1
negative	2955	92.4
Total	3198	100

### 3.3 Concomitant Co‐Allergenic Reactions

Of those who had allergic positive (+ or ++) reactions to 5% sodium benzoate in pet, co‐allergenic responses were seen in 15.4% to benzoic acid and 4.8% to *Myroxylon pereirae* (Table 3). Contrarywise, of those with an allergic response to benzoic acid, 9.5% also reacted to sodium benzoate, but only 2% of *Myroxylon pereirae* reactors showed an allergic test to sodium benzoate. No patients had allergic positives to both benzyl alcohol and sodium benzoate, although one had ?+ to both and one had irritant reactions to both—696 were negative to both these allergens (Table 4). Benzoic acid and benzyl alcohol seem to be able to induce a clinical picture similar to that with sodium benzoate [13].

**TABLE 3:** The allergic co‐reactivities of subjects with + or ++ reactions to sodium benzoate, to 5% benzoic acid in pet, 10% benzyl alcohol in sof, and 25% *Myroxylon pereirae* in pet, in numbers (+, ++ and +++ aggregated to positive (pos), doubtful (?+), irritant (IR) and negative (neg) combined as negative).

		Benzoic acid		
		pos	neg	total
Sodium benzoate	pos.	2	11	13
	neg.	19	781	800
	Total	21	792	813

		Benzyl alcohol		
		pos	neg	total
Sodium benzoate	pos.	0	21	21
	neg.	3	1376	1379
	Total	3	1397	1400

		Myroxylon pereirae		
		pos	neg	total
Sodium benzoate	pos.	1	20	21
	neg.	50	1342	1392
	Total	51	1362	1413

**TABLE 4:** Concomitant reactions to 5% sodium benzoate in pet. and 5% benzoic acid in pet. without aggregation.

		Benzoic acid					
		+	++	?+	IR	neg.	Total
Sodium benzoate	+	2	0	0	0	10	12
	++	0	0	0	0	1	1
	?+	0	0	1	0	15	16
	IR	1	0	0	1	29	31
	neg.	18	0	8	31	696	753
	Total	21	0	9	32	751	813

### 3.4 Relevance

Clinical relevance was identified in 67% (38 of 57) of + or ++ reactors, and in 36% (19 of 53) who showed a ?+ response. The most common primary contactants for sodium benzoate were rinse‐off products (e.g., soaps, shampoos, in 20 of the 57 +/++ reactors) and leave‐on cosmetics (15/57). Glove use was a concomitant factor in 8 of 57. One possible mechanism is by increasing skin penetration of sodium benzoate encountered through personal care products just used by the individual. Contactants in textiles, dental products (e.g., toothpaste or mouthwash), topical medication, hair cosmetics, plant products, deodorant and oils were identified in small numbers [14].

## 4 Discussion

The findings here of both a significant rate of allergic positives (bakery/cheilitis 1.5%; facial 1.7%; fragrance 2.1%) together with an observed clinical relevance of two‐thirds in those showing + or ++ at 4 days, underlines the importance of including 5% sodium benzoate in pet in these patch test series. However, we identified technical problems with the 5% preparation. The high positivity ratio (96.5%) means that most allergic positives were weak (+), with very few strong (++) and no extreme (+++), and the low reaction index (−0.53) indicates a high rate of doubtful (?+) and irritant reactions. This supports the Informationsverbund Dermatologischer Kliniken data for sodium benzoate of a positivity ratio of 92% and a reactivity index of −0.23, based on 79 046 cases seen from 1996 to 2009—the reason for a difference in the reactivity ratio here is unclear [8]. The 36% clinical relevance in ?+ cases indicates that quite a proportion of true allergic reactions are being missed. Taken together, these data indicate the need for refinement of the patch testing material used to identify sodium benzoate contact allergy. The high rate of irritant reactions is an indicator that studies of patch testing with sodium benzoate at lower concentrations would be beneficial.

Cross reactivity is said to be in play when a threshold for reactivity is around 10% or greater for the related substance [15]. By this definition in this study, cross reactivity seems to exist for sodium benzoate and benzoic acid, since 15.4% of sodium benzoate reactors were also positive for benzoic acid, and 9.5% of benzoic acid reactors were also positive to sodium benzoate. These figures are different from the 42.9% and 6.8% respectively found by Scheman et al., though both lend support to the cross‐reactivity concept [15]. The degree of cross reactivity, however, in our study indicates the necessity to patch test additionally with both sodium benzoate and benzoic acid to maximise the return. Reactions for sodium benzoate reactors with *Myroxylon pereriae* (4.8%) and benzyl alcohol (0%) did not support cross‐reactivity with these preparations.

The issue of the inter‐relationship between the aforementioned allergens raises the phenomenon highlighted by Nijhawan and Jacob, of the ‘whole being greater than the sum of its parts’, the phrase used to describe the synergistic action of two or more substances whose combined effect was greater than the sum of their individual effects [16]. The latter authors found that the combined effect of testing cross‐reactors and constituents simultaneously with the allergen in question—in their instance specifically *Myroxylon pereriae*—aided in evoking a positive allergic response and hence assisting in diagnosis and management. Nijhawan and Jacob specifically mention benzyl alcohol and found that its concurrent application increased identification of *Myroxylon pereriae* or its constituents. They observed that the addition of cross‐reactors and constituents to the patch‐test panel may decrease false negatives and increase the detection of true positive patch‐test reactions, possibly by overcoming a threshold [16]. This is advice worth noting when testing with allergens of uncertain validity, and is relevant to the present case.

It is worth considering the immunological mechanisms involved. Research suggests this to be a type 2 immune contact hypersensitivity response driven by T helper 2 (Th2) cells. In inflamed skin, such as that in atopic dermatitis, recent evidence shows a shift from type‐2 innate lymphoid cells (ILC2) towards type‐3 innate lymphoid cells (ILC3) that can produce the chemo‐attractive cytokines interleukin‐17 (IL‐17) and IL‐26, thus amplifying the immune reaction and thus engendering inflammation [17]. It has been proposed that in this paradigm, ILCs are exhibiting memory‐like properties, responding to antigens without possessing antigen‐specific receptors—in a manner akin to the innate contact hypersensitivity response [18]. Further study in this area may finesse the immunological distinctions between irritant and allergic contact reactions.

On a practical level, when patch testing to benzoic acid, especially if a reaction to a cosmetic substance is being investigated, the short patch test with a reading at 20–30 min should be considered, in addition to the 2 (possibly 3) and 4 days normal protocol [19]. Some authors patch test allergens (in this case the benzoic acid) in duplicate and in two concentrations to ensure a clear picture [20].

Our study has certain limitations. Due to the exigencies of our service, a small number of patients had their readings done on days 3 and 5 rather than days 2 and 4—our practice has not been to routinely make a 7‐day reading unless it was specifically indicated by the 2‐ and 4‐day results. We accept that this is an imperfect situation and may lead some to question the validity of our conclusions. However, we would point out results from the recent publication of Cantwell and colleagues from the Mayo Clinic, Rochester, Minnesota, based on 9 years of data encompassing 42 438 subjects [21]. These authors came to recommend 7‐day readings for metals and possibly for acylates, but not for other allergens—for which they felt that a 5‐day reading was adequate (counting the application day as day 1). On this basis, we feel reasonably confident that our conclusions are intact. An additional potential shortcoming of this investigation is that most of our patients are discharged after being patch tested, so follow‐up information is lacking on avoidance measures or repeated open application tests. Some data are missing from the MOALHFA set. When assessing relevance, products containing benzoic acid were not considered when perhaps they should have been. Type I contact urticarial reactions were not looked for specifically but were not identified on routine history taking.

## 5 Conclusions

In conclusion, we have identified sodium benzoate as a significant allergen, well worth inclusion in a number of patch test series. However, patch testing with it is problematic in view of the propensity of the test preparation to induce weak allergic positive reactions in the allergic population, making for difficulties in assessment and requiring a high degree of suspicion for weak (+) or doubtful (?+) reactors, in whom it may indeed be an allergen of relevance. Lowering the concentration tested to below the current 5% might reduce the high rate of irritancy but would also lessen relevant weak positives, whilst increasing the test concentration may produce stronger allergic positives but would indubitably result in more irritants and, likely, false allergic positives. Nonetheless, some refinement of the test material might resolve these dilemmas.

## Author Contributions

**Nicholas J. Lawrance:** conceptualization, methodology, writing – original draft. **Catherine R. Holden:** conceptualization, methodology, writing – original draft, investigation. **David J. Gawkrodger:** investigation, writing – review and editing.

## Disclosure

The project was approved by the Quality Improvement Department of the Sheffield Teaching Hospitals NHS Foundation Trust.

## Conflicts of Interest

The authors declare no conflicts of interest.

## Data Availability

The data that support the findings of this study are available from the corresponding author upon reasonable request.
